# Supplementary material for: Seminoma and Embryonal Carcinoma Footprints Identified by Analysis of Integrated Genome-Wide Epigenetic and Expression Profiles of Germ Cell Cancer Cell Lines
Source: PLoS One. 2014 Jun 2;9(6):e98330. doi: 10.1371/journal.pone.0098330 (PMC4041891; doi:10.1371/journal.pone.0098330)
Supplement: File S1 — ZIP file containing DMRforPairs output for significant regions. Please start from the html files. (ZIP) [file pone.0098330.s008.zip › figures/36898.pdf]

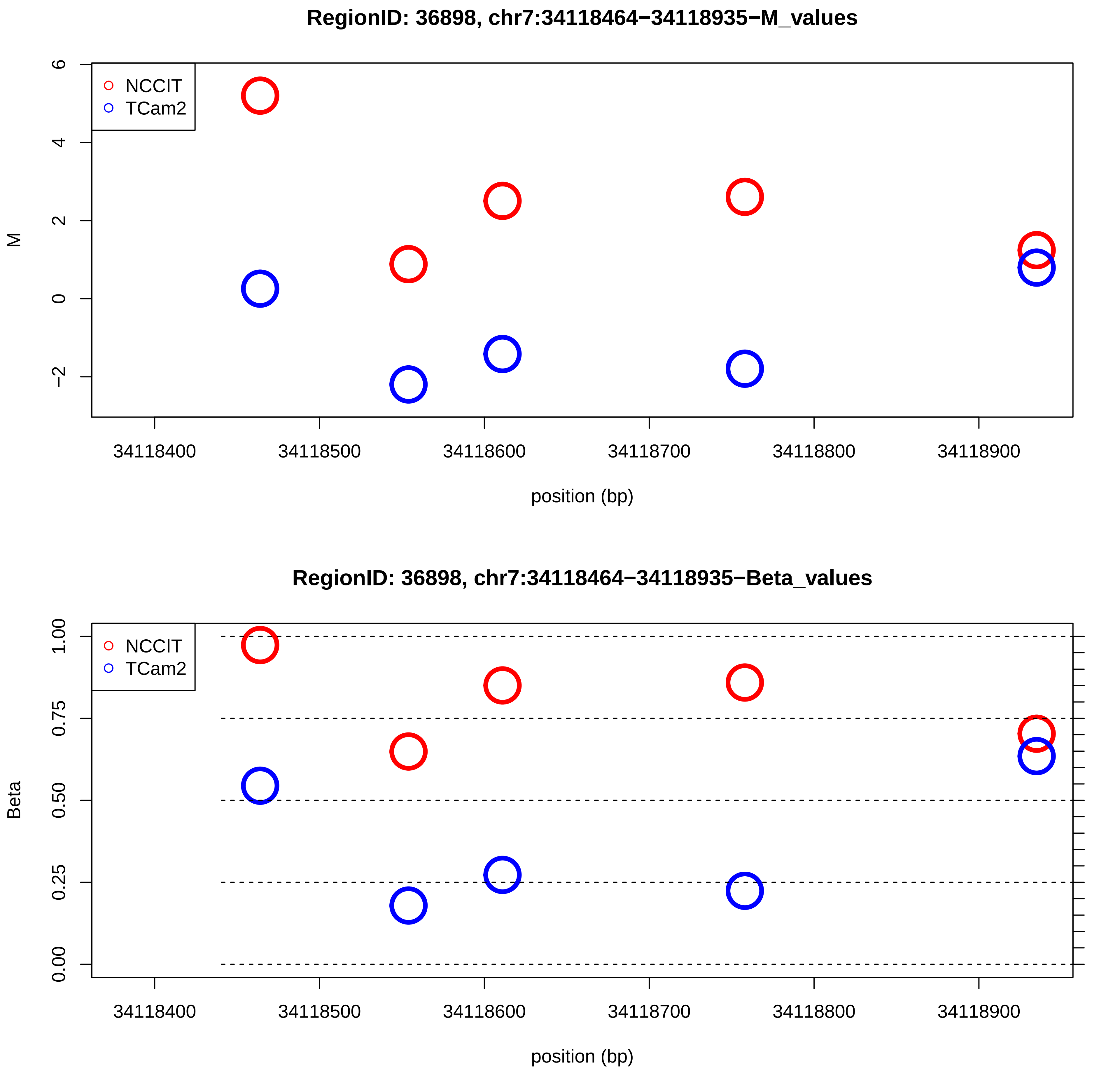

RegionID: 36898, chr7:34118464−34118935−M_values
M
6
4
2
0
−2
NCCIT
TCam2
34118400
34118500
34118600
34118700
34118800
34118900
position (bp)
RegionID: 36898, chr7:34118464−34118935−Beta_values
Beta
1.00
0.75
0.50
0.25
0.00
NCCIT
TCam2
34118400
34118500
34118600
34118700
34118800
34118900
position (bp)
